# Assessing environmental impacts of large centralized wastewater treatment plants with combined or separate sewer systems in dry/wet seasons by using LCA

**DOI:** 10.1007/s11356-020-08038-2

**Published:** 2020-02-20

**Authors:** Siti Safirah Rashid, Yong-Qiang Liu

**Affiliations:** Faculty of Engineering and Physical Sciences, University of Southampton, Southampton, SO17 1BJ UK

**Keywords:** Wastewater treatment plants, Sewer system, Wet and dry seasons, Life cycle assessment, Functional units

## Abstract

Rainfall can affect influent flow rate and compositions of wastewater, and thus further affect wastewater treatment performance and the effluent quality. This study aims to study the influence of rainfall on the environmental impacts of centralized wastewater treatment plants. The correlations between rainfall, and influent flow rate and compositions of wastewater in wet and dry seasons with two sewer systems, i.e. combined and separate sewer systems, were primarily established. Environmental impacts were assessed with life cycle assessment (LCA) to understand the temporal environmental burdens in wet and dry seasons. Functional units as per m^3^ treated wastewater (FU1) and as per kg PO_4_^3^-eq. removed (FU2), respectively, were used to evaluate impacts of wastewater treatment to the environment. Strong correlation between rainfall and the influent flow rate was found in the wastewater treatment plants with either a combined sewer system (with Pearson correlation coefficient *r* at 0.66) or a separate sewer system (with *r* at 0.84), where *r* represents the strength of the association between two variables. The rainfall effect is more obvious on the eutrophication potential and global warming potential than on other environmental indicators while sewer system, i.e. combined or separate, seems not important in the two cases studied. Both wastewater treatment plants (WWTPs) show a lower environmental burden in the wet season than in the dry season partially due to the dilution of wastewater by using FU1. The WWTP receiving high strength wastewater, however, demonstrates higher environmental impacts in the wet season by using FU2 than FU1, due to the less efficient treatment caused by heavy rainfall. Meanwhile, it is found that environmental impacts from the WWTP receiving low strength wastewater have no difference when using either FU1 or FU2. The results indicate that the environmental burdens particularly eutrophication and global warming caused by WWTPs are dependent on the correlations of rainfall intensity with wastewater quantity and quality instead of combined or separate sewer system. This could be used to guide a stricter control of eutrophication in a more sensitive season in more vulnerable receiving waters.

**Electronic supplementary material:**

The online version of this article (10.1007/s11356-020-08038-2) contains supplementary material, which is available to authorized users.

## Introduction

Municipal wastewater treatment plants mainly deal with domestic wastewater, but it is a very common practice worldwide that storm runoff, through a combined sewer system, is combined with domestic wastewater for treatment. During wet weather, the untreated wastewater together with storm runoff could overload wastewater treatment plants (WWTPs), leading to overflow of wastewater directly into receiving waters. Even without overflow, rainfall still can affect environmental impacts from WWTPs by changing wastewater quality, quantity and treatment performance. Life cycle assessment (LCA) is an efficient tool to evaluate environmental impacts from WWTPs. LCA is known as a technique for a holistic environmental assessment of a product or system. Since 1990s, LCA has been applied to the field of wastewater treatment (Corominas et al. 2013). In a study with LCA, Risch et al. (2018) reported that loads from storm events contributed significantly to eutrophication and ecotoxicity of WWTPs in freshwater. In addition, the compositions and strength of wastewater to WWTP change accordingly with the variation of rainfall which could affect wastewater treatment performance and the quality of effluent to the environment. Moderate to strong correlations were observed between rainfall intensity and pollutant concentrations in influent as well as rainfall intensity and volumetric flow rate of wastewater at 24 WWTPs in Georgia state, America, with combined sewer systems (Mines et al. 2006). The square of correlation coefficient, *R*^2^, between flow rate and average monthly rainfall ranged from 0.21 to 0.85, indicating that the flow rates of wastewater to WWTPs with combined sewer systems in different catchment areas were affected by rainfall intensity to different extents (Mines et al. 2007). It is believed that highly pollutant loaded influent in dry season can usually have satisfactory levels of pollutants removal while diluted influent by storm water is prone to cause operational issues (Lorenzo-Toja et al. 2015; Risch et al. 2018), and lower treatment efficiency. In many cases, however, lower effluent pollutant concentrations were reported from WWTPs during wet weather due to the dilution of wastewater (Joel et al. 2017; Li et al. 2017). Wastewater characteristics (e.g. concentrations of pollutants) in influent are one of the most important parameters to affect wastewater treatment efficiency, and effluent quality, leading to different environmental impacts from WWTPs. So far, the vast majority of LCA studies of WWTPs, however, were based on the dry weather conditions without considering rainfall effects, which does not enable a holistic view at the scale of the year with the temporal variability of environmental burdens. This is particularly important to the vulnerable receiving waters as dry weather–based environmental impact assessment might overestimate or underestimate the environmental burdens such as eutrophication and ecotoxicity.

Due to the importance of rainfall effects on flow rate and pollutant concentrations of wastewater influent, treatment performance in WWTPs and pollutant concentrations in effluent, a few of studies evaluated the effects of rainfall on the environmental impacts of wastewater treatment plants. Nevertheless, conclusions from these studies are not consistent. For example, for a Spanish municipal WWTP, Moreira et al. (2004) concluded that the differentiation of wet (humid) and dry seasons for environmental analysis was not necessary because the data variability in each season had turned out to be more significant than the variation caused by rainfall. Lorenzo-Toja et al. (2016), however, found that Atlantic region with the highest rainfall resulted in the least environmental impact when they studied WWTPs with LCA in different regions of Spain with different rainfall intensity (i.e. from 300 mm to > 1000 mm). Results from 3-year data in a WWTP, China, with a subtropical monsoon climate showed five chosen impacts (e.g. abiotic depletion potential (ADP), acidification potential (AP), eutrophication potential (EP), global warming potential (GWP) and photochemical ozone creation potential (POCP) increased almost linearly with monthly precipitation when the monthly precipitation was below 200 mm/month (Li et al. 2017). This result indicates higher environmental burdens in the wet season. These contradictory results about rainfall effects (i.e. no impact, positive impact or negative impact) indicate that some key factors that might influence environmental impacts by LCA are still not fully understood. Some possible factors are identified as below. The rainfall effects on WWTPs should be closely related to how much it can cause the changes of influent characteristics including flow rate and concentrations instead of the absolute precipitation amount. Secondly, one of the most important factors affecting the efficiency of WWTPs has been revealed to be the characteristic of the influent particularly wastewater strength (Lorenzo-Toja et al. 2015). Rainfall during wet weather does not only affect wastewater strength in influent and effluent by dilution but also treatment performance. These in turn affect environmental impacts from WWTPs. Thirdly, choosing different functional units might lead to different LCA results on the study of rainfall as influent wastewater quality is changed by rain, but not reflected by some functional units. Per m^3^ treated wastewater is a mostly used functional unit for LCA analysis of WWTPs. However, it is argued that per m^3^ treated wastewater could not reflect the influent quality or wastewater treatment efficiency in WWTPs (Corominas et al. 2013), making the comparison between two systems with different influent quality or different wastewater treatment efficiency difficult. Instead, per kg pollutant removed such as per kg of chemical oxygen demand equivalent (COD-eq.) removed (Wang et al. 2018) or per kg of phosphate (PO_4_^3−^eq.) removed (Rodriguez-Garcia et al. 2011) could be a better functional unit when considering different influent quality or treatment efficiency for the comparative studies. Per population equivalent (P.E.) could also be considered when reflecting the difference of flow rate of influent and the associated load (Gallego et al. 2008; Kalbar et al. 2013). The comparison between two different functional units, e.g. per m^3^ treated wastewater and per kg PO_4_^3−^eq. removed, resulted in contrasting results in terms of main environmental impacts (Rodriguez-Garcia et al. 2011), highlighting the importance of the selection of functional unit in different scenarios. It is thus suggested that LCA studies on WWTPs are preferably carried out using more than one functional unit to deepen understanding of the system under study and to avoid misleading conclusions (Zang et al. 2015). For the study of rainfall effects on the environmental burdens from WWTPs, assessing different functional units is important because the influent quality and quantity changed by rainfall could affect the treatment performance due to the dilution of the influent and the disturbance to biological treatment.

This study aims to investigate the influence of rainfall on the environmental impacts of WWTPs by using LCA in two scenarios, i.e. large centralized WWTPs with high strength wastewater and low strength wastewater, respectively, but with similar rainfall effects on influent flow rate. Meanwhile, different functional units would be studied to evaluate their influence on LCA results in the scenarios with/without rainfall.

## Materials and methods

### The selection and description of two case studies

A pre-screening assessment by us found that the correlation coefficients between monthly rainfall intensity and influent flow rate of wastewater to two WWTPs, i.e. a Malaysian Sewage Treatment Plant (MSTP) in Penang, Malaysia, and Millbrook Wastewater Treatment Work (MWTW), in Southampton, the UK, are similar. In addition, the strength of wastewater in MSTP and MWTW are distinctive. Thus, these two WWTPs were selected to study the effects of rainfall on the environmental impacts of WWTPs with different wastewater strength.

**Fig. 1:**
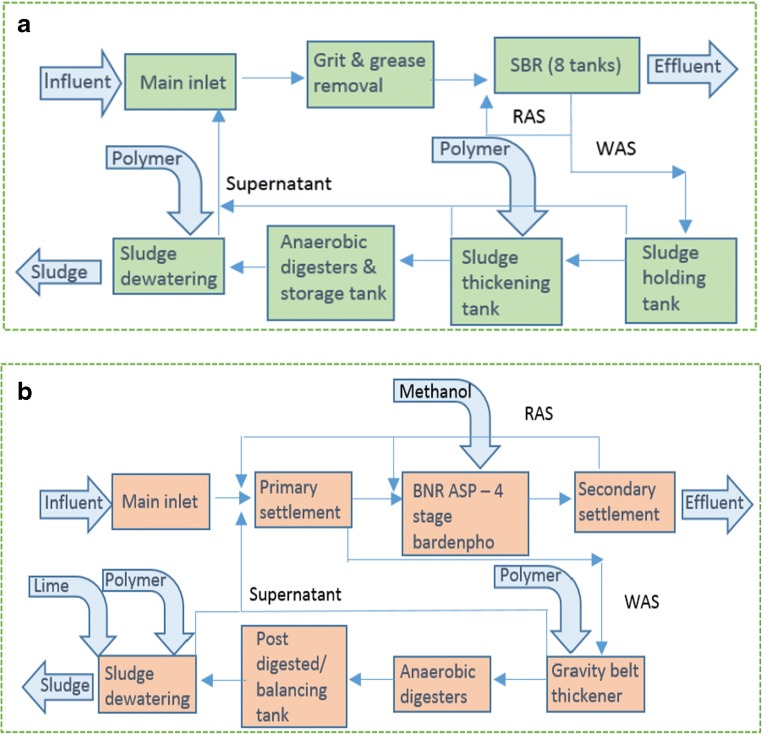
The system boundary of **a** Malaysian STP and **b** Millbrook WTW in this study. (SBR sequencing batch reactor, BNR-ASP biological nutrient removal-activated sludge process, RAS return activated sludge, WAS waste activated sludge)

MSTP receives domestic wastewater of 800,000-population equivalent (PE) with a flow rate varying between 111,191 and 149,584 m^3^/day throughout the year 2016. Wastewater enters into MSTP from a separate sewer system. MSTP mainly consists of grit and grease screening, sequencing batch reactor for pollutant removal, gravity belt thickener, anaerobic sludge digester and biosolids dewatering. This type of WWTP is widely used in Malaysia and is considered as a typical wastewater treatment plant. The treated water is discharged into the river nearby, while the sludge produced is sent to a landfill located 47 km away. The operation data in 2016 was used in this study. Daily rainfall data in 2016 was retrieved from the Malaysian Meteorology Department in the MSTP catchment area. The average monthly rainfall and temperature data from the year 2010 to 2016 were obtained from the web source: (www.worldweatheronline.com) for the comparison of the seasonal pattern. MWTW with a combined sewer system has a wastewater treatment capacity for 140,000 PE with a flow rate varying between 35,028 and 49,563 m^3^/day throughout the year 2017. This facility includes primary settlement, Bardenpho process for COD and nitrogen removal, secondary settlement, sludge thickening, dewatering and anaerobic digestion incorporated with biogas collection and energy recovery systems. Methanol is dosed as an external carbon source for denitrification, and polymer is used for thickening and centrifuges while lime is used for sludge disinfection. Biosolids after digestion are sent for various land application. Rainfall and temperature data in the year 2013 to 2017 in Southampton was obtained from the weather website (www.worldweatheronline.com). Figure 1 shows the schematic diagrams of MSTP and MWTW.

Table 1 provides wastewater quality and quantity entering the MSTP in the year 2016 and MWTW in the year 2017. The storm flow (maximum) was 1.36 times of the dry weather flow (minimum) in MSTP, which is similar to 1.42 times in MWTW. Influent mass load (kg/month) entering the MSTP and MWTW in the dry seasons were at least 1.6 and 2.2 times, respectively, of that in wet season. The ratios of mass and pollutant concentration in dry to wet season were higher in MWTW probably because various pollutants were carried in by stormwater runoff to the treatment plant through a combined sewer system in rainy days (Li et al. 2017). According to the review of wastewater strength in developed countries and developing countries (Gallego-Schmid and Tarpani 2019), average influent BOD, COD and SS concentration is 251, 551 and 252 mg/L, respectively, in developed countries, higher than those in developing countries such as 209, 410 and 190 mg/L, respectively. In addition, BOD, COD, SS, N and P vary in wide ranges in either developed or developing countries. The strength of wastewater into each WWTP in our study falls well within the pollutant concentration range in developing and developed countries (Gallego-Schmid and Tarpani 2019), respectively, but the strength of wastewater into MWTW, UK, is at the upper limit of the range in developed countries while the strength of wastewater into MSTP, Malaysia, is at the bottom limit of the range in developing countries. These two WWTPs are thus ideal for the study on if wastewater strength plays a role when studying rainfall effects on environmental impacts.

**Table 1:** The fluctuations of pollutant concentration, mass load and flow rate of wastewater into Malaysian STP (year 2016) and Millbrook WTW (year 2017), respectively

	Concentration (mg/L)			Mass (kg/month)		
Malaysian STP	Maximum	Minimum	Ratio	Maximum	Minimum	Ratio
Flow (m^3^/month)	4.55 × 10^6^	3.34 × 10^6^	1.36	32 gBOD/head	16 gBOD/head	2.00
BOD_5_	141.00	69.00	2.04	5.25 × 10^5^	2.59 × 10^5^	2.03
COD	530.40	229.50	2.31	1.16 × 10^6^	4.65 × 10^5^	2.49
TSS	150.00	49.00	3.06	5.82 × 10^5^	1.75 × 10^5^	3.33
TN	32.50	20.00	1.63	1.30 × 10^5^	8.10 × 10^4^	1.60
Millbrook WTW	Maximum	Minimum	Ratio	Maximum	Minimum	Ratio
Flow (m^3^/month)	1.49 × 10^6^	1.05 × 10^6^	1.42	100 gBOD/head	19 gBOD/head	5.26
BOD_5_	459.00	91.8	5.00	5.00 × 10^5^	1.21 × 10^5^	4.13
COD	1215.00	327.50	3.71	1.41 × 10^6^	4.35 × 10^5^	3.24
TSS	625.00	168.00	3.72	8.04 × 10^5^	1.96 × 10^5^	4.10
TN	71.00	35.40	2.00	9.13 × 10^4^	4.13 × 10^4^	2.20

*BOD5* 5-day biochemical oxygen demand, *COD* chemical oxygen demand, *TSS* total suspended solids, *TN* total nitrogen

### Correlation analysis of wastewater indicators

In this study, 12 months of operation data in MSTP in the year 2016 and in MWTW in the year 2017 were evaluated with a statistical method to correlate different parameters (Mines et al. 2007; Li et al. 2017). Average monthly rainfall was plotted against average monthly influent flow rate in both plants. Trend lines and the square of correlation coefficient *R*^2^ were determined using linear regression analysis in both plants. In addition, the Pearson coefficient’s correlation analysis between rainfall intensity, sewage temperature, power consumption, volumetric flow rate and other pollutant parameters in influent at a monthly basis was conducted using SPSS software v24.

### Life cycle analysis

#### Goal and scope

The goal of this study is to investigate and compare the effect of rainfall from dry season and wet season on the environmental impacts from large centralized municipal wastewater treatment plants with different influent wastewater strength. Since this study focuses on rainfall effect on the life cycle environmental impacts from WWTP operation, construction and demolition stages as well as landfilling sludge are not considered because they are same regardless of rainfall. However, transport of sludge to landfill was included. For this selection, ‘gate-to-gate’ analysis is adopted which begins with the wastewater influent physically entering into WWTPs, and ends with the effluent discharged into water bodies and transport of biosolids to landfill. The illustrated system boundary for this LCA–WWTP study is shown in Fig. 1**.** In general, the system boundary is limited to wastewater treatment operations with wastewater flow rate and pollution loads in a foreground system, and energy and chemical consumption (e.g. electricity and chemical production) in a background system.

#### Functional unit

1 m^3^ of treated wastewater was used as a functional unit first, which is widely adopted for life cycle impact assessment (LCIA) in WWTPs (Piao et al. 2016; Lorenzo-Toja et al. 2016; Rahman et al. 2016; El-Sayed et al. 2010; Niero et al. 2014). It is believed that the functional unit as per m^3^ of treated wastewater, however, does not consider the change of wastewater flow rate to WWTPs (Piao and Kim 2016) or wastewater treatment efficiency. Therefore, functional unit 2 (FU2) defined as 1 kgPO_4_^3^-eq. removed was used as well for a better comparison with the change of wastewater flow rate. FU2 was also used by Rodriguez-Garcia et al. (2011) and Comas Matas (2012). The eutrophying substances, i.e. chemical oxygen demand (COD), total nitrogen (TN) and total phosphorus (TP) in wastewater were converted to kgPO_4_^3^-eq. using the characterization factor from eutrophication potential impact category as defined in the CML-IA baseline v3.04 methodology.

#### Life cycle inventory

The operation data of MSTP in 2016 and the data of MWTW in 2017 were considered in this study. The life cycle inventory consists of monthly electricity consumption, monthly volume of wastewater treated and daily influent and effluent characteristics. The life cycle inventory (LCI) consists of following parameters: (1) inputs of resources including energy and chemical consumed for wastewater treatment and sludge treatment as well as sludge transportation; (2) influent pollutants as inputs and effluent pollutants as outputs; (3) gas emissions from the plant as outputs, which mainly include CO_2_, CH_4_ and N_2_O. They were calculated according to the Intergovernmental Panel on Climate Change guideline (IPCC 2006) based on the 100-year time horizon. Direct N_2_O was mainly generated from biological nitrogen removal process and CH_4_ was from anaerobic wastewater and/or sludge treatment (Masuda et al. 2015). All inventory data are provided in Table 2 (FU1) and in Table S3 in the supplementary (FU2). Background data were obtained from the Ecoinvent v3.3 database as described below:

- Electricity production: Malaysia and the UK were selected from the Ecoinvent v3.3 database.
- Chemical production: Data on the processes of methanol and lime were selected from the ELCD and Ecoinvent v3.3 database. For polyelectrolytes, a similar production process for acrylonitrile was taken from the Ecoinvent v3.3 (Rodriguez-Garcia et al. 2011; Lorenzo-Toja et al. 2016).
- A lorry with a capacity of 3.5–7.5 metric ton was selected as transport vehicle for the disposal of sludge and wastes produced from both WWTPs. Chemical transportation to the site is excluded due to small proportion to environmental impact (Lorenzo-Toja et al. 2016) with less than 5% emission compared to the sludge transportation value (Rodriguez-Garcia et al. 2011).

**Table 2:** Life cycle inventory (LCI) data in Malaysian STP and Millbrook WTW according to per functional unit (i.e. 1 m^3^ of treated wastewater)

Inventory components	Malaysian MSTP		Millbrook MWTW		Unit/m^3^
	Dry season^A^	Wet season^B^	Dry season (summer)^C^	Wet season (winter)^D^	
1. Electricity consumption	2.58E−01 ± 9.0E−2	2.38E−01 ± 1.7E−2	6.11E−01 ± 5.9E−2	4.86E−01 ± 4.2E−2	kWh
2. Transportation of sludge and waste	6.48E−03 ± 1.9E−6	6.47E−03 ± 4.7E−7	2.70E−02 ± 9.0E−3	2.62E−02 ± 8.0E−3	t.km
*Polymer consumption*					
3. Methanol	–	–	7.12E−03 ± 4.0E−3	6.93E−03 ± 3.0E−3	kg
4. Polyelectrolyte	5.15E−04 ± 1.8E−8	5.15E−04 ± 1.3E−8	3.62E−03 ± 7.0E−3	3.52E−03 ± 6.8E−3	kg
5. Lime	–	–	8.19E−02 ± 8.4E−3	7.97E−02 ± 7.5E−3	kg
*Emission to air*					
6. Carbon dioxide (biogenic)^E^	9.69E−02 ± 7.8E−3	8.17E−02 ± 1.5E−3	3.84E−01 ± 5.5E−2	3.07E−01 ± 4.1E−2	kg
7. Methane, CH_4_	1.11E−03 ± 3.3E−4	1.09E-03 ± 8.6E−4	2.40E−03 ± 9.6E−4	2.00E−03 ± 8.2E−4	kg
8. Dinitrogen monoxide, N_2_O	4.87E−04 ± 7.4E−5	4.40E−04 ± 2.1E−5	5.40E−04 ± 8.3E−5	5.10E−04 ± 7.1E−5	kg
*Emission to water*					
9. Total COD	5.10E−02 ± 8.0E−3	4.28E−02 ± 1.0E−2	4.61E−02 ± 1.0E−2	3.20E−02 ± 9.0E−1	kg
10. Total nitrogen	1.08E−02 ± 2.5E−3	7.63E−03 ± 2.2E−3	9.00E−03 ± 2.5E−3	6.95E−03 ± 1.8E−3	kg
11. Total phosphorus^F^	2.20E−03	1.10E−03	1.1E−03	8.00E−04	kg
*Combined sewer overflow* (*CSO*)					
12. Total COD^G^	x	x	x	4.34E−01	kg
13. Total nitrogen ^G^	x	x	x	2.55E−02	kg
14. Total phosphorus ^G^	x	x	x	4.00E−03	kg

^A^From January to March 2016

^B^From September to November 2016

^C^From June to July 2017

^D^From January to February 2017

^E^Carbon dioxide emission from the biological process in WWTP is considered biogenic origin by IPCC guideline and was not included in the LCA analysis (IPCC Guidelines for National Greenhouse Gas Inventories 2006) (IPCC Guidelines for National Greenhouse Gas Inventories 2006)

^F^1 set of TP data

^G^1 set of inventory data from Millbrook WTW management for CSO

#### Life cycle impact assessment

Life cycle impact assessment (LCIA) was conducted with the characterization factors from CML-IA baseline v3.04 methodology. As wastewater treatment plants mainly generate climate change-related impacts and environmental quality issues (Renou et al. 2007), seven characterization impact categories such as eutrophication potential (EP), ozone layer depletion potential (ODP), freshwater ecotoxicity potential (FEP), human toxicity potential (HTP), global warming potential (GWP), abiotic depletion (fossil fuel) potential (ADFP) and acidification potential (AP) were chosen as the main assessment categories.

#### Life cycle interpretation

The LCA results were interpreted to assess the contribution of each component in the inventory to each environmental impact category. Sensitivity analysis was conducted to evaluate how the change of inventory data affects LCA results (impact categories). In this way, the effects of the accuracy of inventory data could be evaluated.

## Results and discussion

### Multivariate correlation between various parameters of wastewater in two WWTPs

**Fig. 2:**
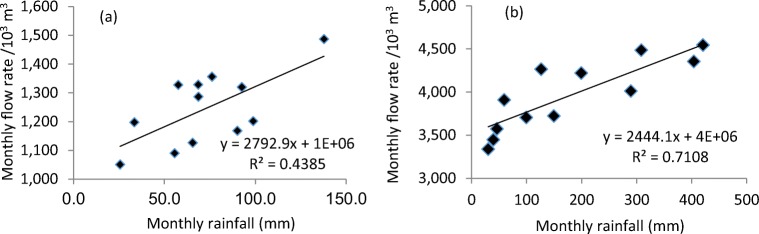
The linear relationship between the average monthly influent volumetric flow rate and the average monthly rainfall intensity for **a** Millbrook WTW and **b** Malaysian STP

Rainfall affects the wastewater flow rate to WWTP particularly with a combined sewer system receiving storm runoff. It can further affect operation in WWTP and quality of effluent to water bodies. A positive linear relationship between the average monthly rainfall intensity and the average monthly influent flow rate into MWTW, Southampton, UK, with a combined sewer system was found as shown in Fig. 2a**.** This is plausible as the high rainfall intensity directly results in the storm runoff into the sewer system, and thus increases the influent flow rate. This result is consistent with those reported in other geographical areas with combined sewer systems. For example, Li et al. (2017) reported a linear relationship between influent flow rate to WWTP and rainfall precipitation with a combined sewer system in Yangtze, Eastern China, where the average yearly precipitation is 1100 mm, comparable with 879 mm in Southampton, UK, in this study. Both WWTPs have similar P.E., e.g. around 186,000–200,000. However, the influent flow rate to WWTP increases by 1480 m^3^ per mm precipitation in the Yangtze, China, while by 2793 m^3^/mm, twofold higher, in this study to MWTW in Southampton, UK. Mines et al. (2006) correlated the rainfall intensity and influent flow rates to 24 WWTPs with combined sewer systems in Georgia state, America, and found similar linear relationships, but the slopes of regression lines range from 540 to 8100 m^3^/mm precipitation in different locations. This is mainly because that the change in flow rates to WWTP caused by rainfall with a combined sewer system relies on both the precipitation amount and hydrogeologies e.g. soil condition for filtration (Metcalf and Eddy 2004), sewer pipe conditions, runoff from the city and the catchment area. The increase rates in influent rate by rainfall to WWTPs with a combined sewer system in different catchments vary, but a linear relationship can well describe the effects of precipitation on the flow rate of influent to WWTP.

For WWTPs with a separate sewer system, a general impression is that rainfall should not cause much change in wastewater flow rate because storm runoff is collected separately. Thus, there lacks studies on the rainfall effects on influent flow rate to WWTPs with separate sewer systems. In this study, however, a linear relationship was established as well between rainfall intensity and flow rate to MSTP, Penang, with a separate sewer system (Fig. 2b), and a similar increasing rate as MWTW in Southampton, UK, with a combined sewer system was found. This contrasts with the general impression that rainfall does not cause much flow rate change to WWTP with a separate sewer system, indicating the complexity of the actual situation with regard to the effect of rainfall intensity on the influent flow rate to WWTPs. In this study, the precipitation in MSTP, Penang, is 2200 mm yearly, which is much higher than 879 mm in the catchment area with MWTW, Southampton. It is thus speculated that water saturation in the soil in Penang, Malaysia, might be higher, leading to more infiltration to the sewer system although it is meant to collect domestic wastewater only. The investigation on the specific reasons for this is beyond the scope of this study, but results here clearly suggest that combined or separate sewer system is not the only decisive factor to determine the effect of rainfall on influent flow rate to WWTPs. To the best of our knowledge, the findings here about rainfall effect on influent flow rate to a WWTP with a separate sewer system are reported for the first time. The comparison of rainfall effect on influent flow rate to WWTPs with a separate sewer system and a combined sewer system in two locations were investigated for the first time, and similar results were obtained. This highlights the necessity to study the rainfall effect on WWTPs even with a separate sewer system.

The great dependence of influent flow rate on rainfall can lead to the changes of wastewater quality (pollutant concentrations) and quantity (flow rate), thus further affects the environmental impact of WWTPs due to the changed power consumption for pumping and aeration, and treatment performance. To understand the relationship between different parameters, the correlations between rainfall, temperature, power consumption and wastewater influent characteristics were carried out and the results are shown in Table 3. When the Pearson correlation coefficient, *r*, moves from 0 to ± 1.0, the correlation becomes stronger. From Table 3, a strong correlation between rainfall and the influent flow rate was found with a Pearson coefficient of 0.84 for MSTP, Penang, with a separate sewer system and 0.66 for MWTW, Southampton, with a combined sewer system. Like linear regression, the correlation is even stronger in MSTP, Penang, with a separate sewer system, which is probably due to higher rainfall intensity and larger catchment area in Penang, Malaysia. In addition, there is a moderate correlation between rainfall intensity and power consumption in MSTP with *r* as 0.54 but only 0.07 in MWTW. It is believed that a weak correlation between rainfall and power consumption for MWTW was from lower precipitation in Southampton at an average monthly of 78.5 mm.

**Table 3:** Pearson correlations between average monthly sewage temperature, rainfall, the flow rate of wastewater, power consumption and influent pollutant concentrations and mass loads in Malaysian STP and Millbrook WTW

Malaysian STP	Temperature	Rainfall	Inflow	Power	BOD_5_m	TCODm	TSSm	TNm
Temperature	1.00	0.02	0.21	0.33	0.28	0.21	− 0.18	0.07
Rainfall	0.02	1.00	*0.84*	*0.54*	0.26	0.42	0.28	*0.58*
Inflow	0.21	*0.84*	1.00	0.44	0.13	0.23	0.06	*0.51*
Power	0.33	*0.54*	0.44	1.00	*0.81*	*0.82*	*0.69*	*0.80*
BOD_5_c	0.16	− 0.15	− 0.34	*0.57*	*0.89*	*0.76*	0.43	0.32
TCODc	0.15	0.11	− 0.13	*0.69*	*0.89*	*0.93*	*0.54*	0.46
TSSc	− 0.25	0.03	− 0.22	*0.54*	0.43	0.46	*0.96*	0.46
TNc	− 0.11	0.07	− 0.14	*0.60*	*0.61*	*0.58*	*0.71*	*0.78*
Millbrook WTW	Temperature	Rainfall	Inflow	Power	BOD_5_m	TCODm	TSSm	TNm
Temperature	1.00	− 0.40	− 0.37	*0.61*	0.10	0.5	*0.55*	0.30
Rainfall	− 0.40	1.00	*0.66*	0.07	0.25	0.03	− 0.09	0.06
Inflow	− 0.37	*0.66*	1.00	− 0.03	0.17	− 0.02	− 0.05	0.25
Power	0.61	0.07	− 0.03	1.00	0.48	*0.69*	*0.57*	0.41
BOD_5_c	0.24	0.02	− 0.14	0.47	*0.95*	*0.61*	0.37	0.34
TCODc	0.56	− 0.12	− 0.24	*0.64*	*0.55*	*0.97*	*0.91*	*0.79*
TSSc	0.60	− 0.20	− 0.23	*0.56*	0.33	0.93	*0.98*	*0.85*
TNc	0.47	− 0.21	− 0.17	0.43	0.36	*0.90*	*0.95*	*0.91*

Values in italics are the correlation values that are higher than ± 0.5

Inflow = influent flow rate, power = electricity consumption, m = mass load, c = concentration

Both plants exhibited negative correlations between influent flow rate, and influent BOD_5_, TCOD, TSS and TN concentrations, indicating a dilution of wastewater by rainfall. This result is in agreement with the findings reported for 24 WWTPs in the USA mainly with combined sewer systems by Mines et al. (2006), who found low to moderate negative correlation between influent flow rate and concentrations of BOD and TSS in the influent. Although rainfall dilutes wastewater in terms of pollutant concentrations, the correlations between rainfall and pollutant mass load (e.g. kg/day) in both MSTP and MWTW are mostly positive. This suggests increased total pollutant mass loads in rainy days, especially in MSTP, due to the pollutants taken in by runoff, which increases the treatment burdens to WWTPs. The correlation between influent flow rate and mass loads of pollutants in MSTP with a separate sewer system was relatively weaker probably due to pollutant filtration by soil before infiltration. These results further indicate the complexity of the correlation between influent flow rate and pollutants (Nesmerak and Blazkova 2014).

In addition, mass loads and pollutant concentrations in influent were highly correlated to the energy consumption in both plants. In MSTP, the correlations were high between power consumption and pollutant loads of BOD_5_m, TCODm, TSSm and TNm with *r* as 0.81, 0.82, 0.69 and 0.8, respectively, while there were relatively moderate values of correlation, e.g. *r*, between 0.41 and 0.69 in MWTW. The power consumption in WWTPs is not fixed the year around, and WWTP uses more energy when it deals with higher pollutant mass loads. It seems that MSTP with bigger capacity (i.e. for an average of 588,000 PE) is more affected. Both plants also exhibited a positive moderate correlation between power consumption and influent pollutant concentrations (mg/L), which further proved the good correlation between power consumption and the pollutant characteristics. The correlation between influent flow rate and power consumption was moderate with *r* of 0.44 in MSTP, while it is only 0.03 in MWTW. With the high rainfall intensity in the MSTP catchment area, the treatment plant consumed higher energy with higher inflow while there was little power consumption change with the inflow change in MWTW. Finally, it is found that influent flow rates correlate negatively to the effluent quality in both MSTP and MWTW (Table S1 in the supplementary), suggesting that the reduction in the concentrations of pollutants in effluent is also from the dilution by rainfall. These results indicate that for either combined or separate sewer system, rainfall does affect wastewater influent flow rate, wastewater influent and effluent quality and power consumption, which further influence the overall environmental impact from WWTPs. Therefore, using one set of data from industry-standard simulation software or from short-period sampling to do static environmental impact assessment with LCA might cause some bias. Thus, it is very necessary to split the whole year as a wet and dry season to see how rainfall in wet and dry seasons with different sewer systems affects wastewater treatment and environmental impact with real dynamic data to provide a basis for further methodology development and validation, as well as the improvement of the LCA practice.

### Rainfall effects on wastewater quality, energy and chemical consumption in two WWTPs

The monthly rainfall intensity versus months in 2016 was plotted to identify wet and dry seasons in MSTP catchment area (Fig. S1 in the supplementary), from which dry season was identified from January to March with the lowest rainfall intensity while wet season from September to November. To validate the consistency of wet and dry seasons over years, the average rainfall intensity from 2010 to 2016 was further analysed to identify wet and dry seasons. The results from 2010 to 2016 are consistent with the year 2016’s rainfall pattern, indicating that 2016 is a year with a typical dry season and wet season. An earlier study on Penang in the year 2000 by Ahmad Jailani (2004) showed the same wet and dry seasons. Similarly, the monthly rainfall pattern in 2017 was compared with that from the year 2013 to 2017 (Fig. S2 in the supplementary) in the MWTW catchment area, and June to July was identified as dry season (summer as well) while January to February is wet season (winter as well).

Table 4 shows the comparison between dry and wet seasons in terms of influent and effluent pollutant concentrations and other parameters in MSTP and MWTW. Higher flow rate and power consumption were found in the wet season than in the dry season at MSTP (Table 4). A 20.1% increase in the flow rate in the wet season was found compared to the dry season at MSTP although a separate sewer system is used, while the flow rate in MWTW with a combined sewer system only increased by 11.2% in the wet season. This highlights that the rainfall effect on wastewater flow rate depends on not only the type of sewer system but also rainfall intensity and other factors.

**Table 4:** Comparison of average monthly parameters, influent and effluent pollutant concentrations in Malaysian STP and Millbrook WTW

	Malaysian STP			Millbrook WTW			
	Dry season^A^	Wet season^B^	Difference (%)^E^	Dry (summer season)^C^	Wet (winter season)^D^	Difference (%)^E^	CSO^F^
Rainfall (mm)	43.0 ± 14.8	378.0 ± 60.5	88.6	62.2 ± 14.4	84.4 ± 32.2	26.2	
Flow rate (m^3^)	3.6E+06 ± 3.0E+05	4.5E+06 ± 9.8E+04	20.1	1.2E+06 ± 1.3E+05	1.3E+06 ± 1.3E+05	11.2	4.8E+04/day
Power consumption (kWh)	9.2E+05 ± 4.5E+04	1.1E+06 ± 5.9E+04	13.8	7.2E+05 ±2.4E+04	6.5E+05 ± 2.6E+04	− 10.0	
Sewage temperature (°C)	21.5	20.2	6.1	20.5 ± 2.2	12.4 ± 1.3	39.5	
*Influent*							
BOD (mg/L)	86.0 ± 11.5	77.7 ± 8.5	9.7	327.5 ± 186.0	213 ± 36.1	35.0	161.0
COD (mg/L)	178.7 ± 65.0	177.3 ± 40.6	0.7	1157.5 ± 81.3	495.5 ± 39.6	57.2	434.0
TSS (mg/L)	96.7 ± 27.9	79.7 ± 13.1	17.6	595.3 ± 42.1	259.8 ± 22.3	56.4	360.0
TN (mg/L)	23.5 ± 2.2	22.3 ± 0.7	5.0	70.3 ± 1.0	47.6 ± 1.2	32.3	25.5
TP (mg/L)	4.0 ± 0.08	3.3 ± 0.07	17.5	6.3	4.2	12.8	4.0
*Effluent*							
BOD (mg/L)	8.9 ± 1.6	4.7 ± 2.4	47.2	8.4 ± 3.1	3.7 ± 0.1	56.0	161.0
COD (mg/L)	51.0 ± 23.5	42.8 ± 10.2	16.1	46.1 ± 3.7	32 ± 0.1	30.6	434.0
TSS (mg/L)	21.2 ± 9.9	14.9 ± 8.0	30.0	7.5 ± 0.2	6.5 ± 2.5	13.3	360.0
TN (mg/L)	10.8 ± 4.2	7.6 ± 3.5	29.3	9.0 ± 0.9	6.9 ± 1.1	22.8	25.5
TP (mg/L)	2.2 ± 0.04	1.2 ± 0.04	45.5	1.1	0.8	27.3	4.0

^A^From January to March 2016

^B^From September to November 2016

^C^From June to July 2017

^D^From January to February 2017

^E^Wet season used as a reference

^F^One set of data on combined sewer overflows (crude effluent) at > 6 DWF (dry weather flow)

The influent pollutant concentrations in dry and wet seasons are relatively stable in MSTP while they varied significantly in MWTW from 12.8 to 57.2%. This might be because that wastewater in MSTP has low strength pollutants even in the dry season while the wastewater strength in MWTW is much higher, leading to more susceptibility of influent pollutant concentrations to rainfall’s dilution. Although the higher temperature in summer (dry season) should be more efficient for the biological treatment to produce better effluent in MWTW, the concentrations of effluent pollutants are higher in summer (dry season). This might be due to much higher influent pollutant concentration in dry season. In MWTW with a combined sewer system, the combined untreated sewage with storm runoff during wet season overflows to rivers when influent flow rate is over 6 times of dry weather flow. Table 4 shows a sample of CSO discharge to the water body on 14th February 2017. Although the pollutant concentrations in CSO in MWTW during a storm event are much lower than the influent concentrations due to dilution, pollutant concentrations are still much higher than the effluent in both seasons. This suggests a risk posed by untreated CSO to public health and the environment. Since the data on the discharge amount and frequency of CSO in MWTW are not available, LCA analysis in this study does not include the environmental impact from CSO. In addition, this can facilitate the comparison between two WWTPs with focuses on wet and dry seasons only in this study without considering CSO.

### Seasonal comparison of seven life cycle environmental impact categories in MSTP and MWTW using FU1 (1 m^3^ treated wastewater) and FU2 (eutrophication reduction—1 kg PO_4_^3^-eq)

#### Environmental impact of two WWTPs in wet and dry seasons using FU1

**Fig. 3:**
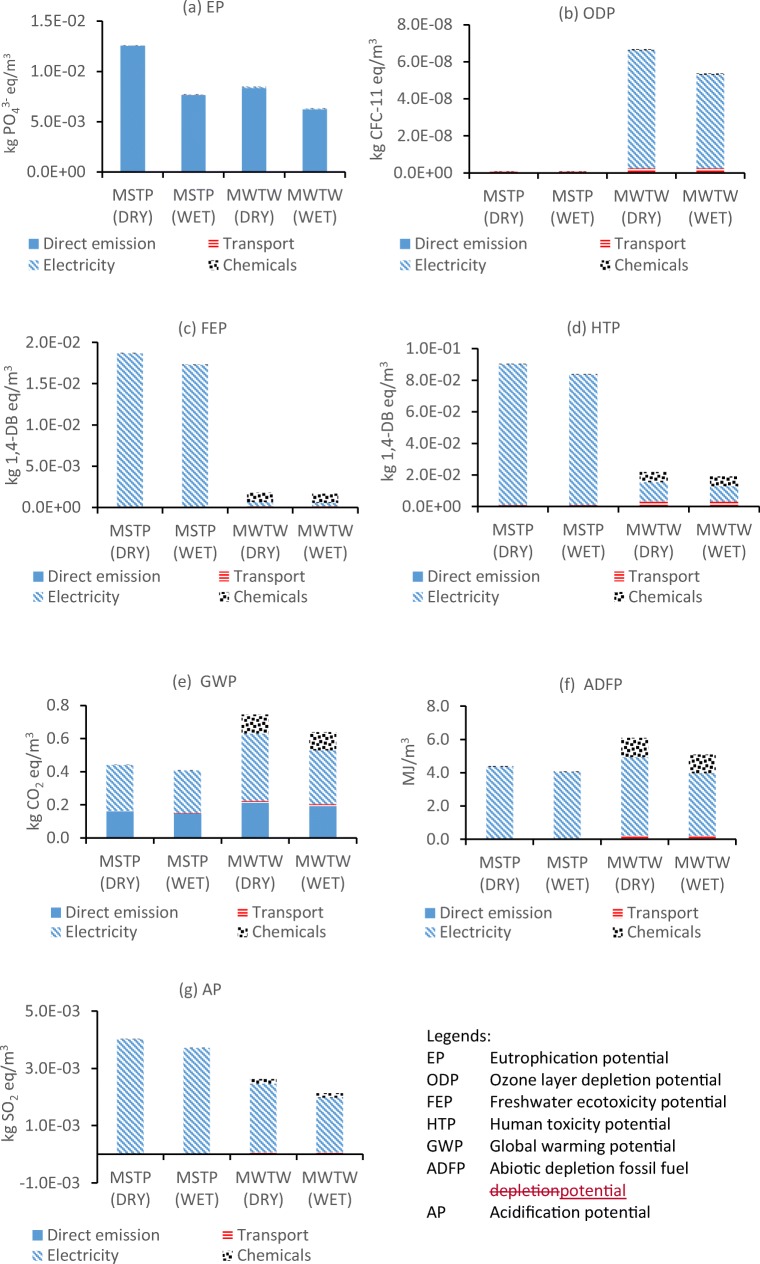
Environmental impact assessment in seven categories at Malaysian STP (MSTP) and Millbrook WTW (MWTW) in both dry and wet seasons by using FU of 1 m^3^ treated wastewater

The environmental impact assessment results of two WWTPs are shown in Fig. 3. It can be seen that environmental impacts of all categories are lower in the wet season than that in the dry season with the difference less than 19.6% except for eutrophication potential (EP), which is 39% lower in MSTP and 25% lower in MWTW in the wet season. This seems straightforward as there are lower pollutant concentrations in the effluent due to the dilution by rainfall (Joel et al. 2017) in the wet season with per m^3^ treated wastewater as the functional unit for comparison. Meanwhile, there are no significant changes in operational conditions such as chemical, power consumption and transportation against the increased flow rate in the rainy season (Piao and Kim 2016). The difference between wet and dry seasons suggests a necessity to do seasonal LCA assessment, especially when considering eutrophication to the environment.

Direct emissions of COD, TN and TP in effluent contribute 99% to EP in both treatment plants during dry and wet seasons. Obviously, to reduce EP impact, it is important to increase TN and TP effluent discharge standards. The current P discharge standard of 1–2 mg/L in the UK (Lesjean et al. 2003) cannot comply with the EU Water Framework Directive 2000/60/EC to reach ‘good’ ecological standard in the country’s watercourses (Howell 2010; Vlachopoulou et al. 2014). Much stricter phosphorus limits such as 0.1 mg/L (for large wastewater treatment works) and 0.5 mg/L (for small sites) are thus set to be imposed in the UK (Jarvie et al. 2006; Howell 2010). This is expected to reduce eutrophication in watercourses greatly. But there is no expected discharge requirement of phosphorus to rivers/streams in Malaysia (DOE Malaysia 2010) in the near future, and it is thus expected that eutrophication in watercourses will still be a problem. EP impact from MWTW in both seasons is lower than that in MSTP although the influent nutrient concentrations in MWTW are 2–3 times higher. This is mainly because MWTW adopts Bardenpho treatment for nitrogen and phosphorus removal to a certain degree while MSTP process is operated only for COD and SS removal. It needs to be pointed out that the eutrophication would be 81% more in MWTW in the senario of combined sewer overflows (CSO) due to the direct raw sewage emission to natural water bodies (Fig. S3 in the supplementary). The other six life cycle impact categories, however, have comparable results between CSO occurrence and the normal winter (wet) condition. This implies that the direct discharge of untreated wastewater to the environment during storm event mainly causes eutrophication. To reduce this impact, the UK is promoting sustainable urban drainage system to reduce CSO frequency or root out the occurrence of CSO from the source (Stovin et al. 2007).

For the other 6 environmental impact categories, energy consumption is the main contributor, dominating in both plants during both seasons. Since the wastewater strength in MWTW is much higher than that in MSTP, and meanwhile MWTW adopts technology for nutrient removal, the electricity consumption in MWTW for treating per m^3^ wastewater is 0.55 kWh while it is only 0.26 kWh in MSTP. Nitrogen removal demands more aeration thus more electricity for nitrification. The environmental impact caused by energy consumption is also related to the energy source for electricity generation. Ninety-three percent of electricity production in Malaysia is depending on fossil fuel while it is only 58% in the UK with the other 42% from renewable and nuclear power.

Electricity consumption accounts for 96% to ozone layer depletion potential (ODP) in MWTW (Fig. 3b). ODP is 19.6% higher in the dry season than the wet season due to 20.5% higher of electricity consumption per functional unit (1 m^3^ treated wastewater) in the dry season. ODP value in MWTW was 99.1% higher than that in MSTP due to 53% higher energy consumption in MWTW per m^3^. This result in MWTW is comparable to those reported by Godin et al. (2011) and Lorenzo-Toja et al. (2016) that high energy consumption per functional unit of 1 m^3^ ranging from 0.4 to 0.7 was used. Both plants have no chemical addition for phosphorus removal; thus, the contribution to ODP is mainly from electricity consumption. It has been reported that the addition of ferric chloride for phosphorus removal or flocculation can contribute to more than 90% of ODP (McNamara et al. 2014; Lorenzo-Toja et al. 2016) because the production of ferric chloride leads to high emission. Thus, based on LCA analysis, appropriate process/chemicals could be chosen in WWTPs to reduce negative environmental impact.

For freshwater ecotoxicity potential (FEP) category (Fig. 3c) and human toxicity potential (HTP) category (Fig. 3d) in both plants, dry and wet seasons do not show an evident difference because of the nearly similar electricity consumption and chemical consumption during these two seasons. But FEP and HTP in MSTP are much higher than those in MWTW and electricity accounts for 99% share while in MWTW chemical consumption contributes to a certain degree. This is mainly because the electricity generation in the UK is less dependent on fossil fuel, which results in smaller FEP and HTP because FEP and HTP are mainly from fossil fuels.

For GWP impact, MWTW is 35% higher than MSTP due to high electricity consumption per m^3^, chemical consumption for denitrification and higher direct emission from high strength wastewater. Regarding seasonality, GWP in the dry season is 7.6% higher in MSTP and 14.2% higher in MWTW, respectively. This difference is mainly caused by the difference in energy consumption per functional unit due to the seasonal difference in influent quality as well as the wastewater strength. The dilution effect from storm runoff is more effective to relatively high strength wastewater in MWTW to result in a less environmental impact in the wet season due to the reduced power consumption and the less direct emission due to the reduced wastewater strength. GWP values in this study ranging from 0.40 to 0.73 kgCO_2_eq/m^3^ are in accordance with those reported by Rodriguez-Garcia et al. (2011), Corominas et al. (2013) and Lorenzo-Toja et al. (2016) with GWP ranging from 0.44 to 0.71 kgCO_2_eq/m^3^. This suggests a consistent GWP range from WWTPs. It has to be pointed out that apart from electricity consumption, direct emission from wastewater treatment processes is also an important contributor to GHG emission. However, it is believed that this direct emission is usually underestimated by the calculation guided by IPCC. Based on the actual measurement on-site, direct emission could contribute up to 71% of the total GHG (Delre et al. 2019). This poses a great challenge to WWTPs to optimize the treatment process to reduce direct emission especially N_2_O from nitrogen removal process and CH_4_ from sewage and sludge handling.

For abiotic depletion (fossil fuel) potential (ADFP) (Fig. 3f), MSTP presents a slightly better result than MWTW because MWTW uses more electricity per m^3^ treated water but lower fossil fuel percentage in the grid. Again, the season difference, i.e. 16.4%, is more obvious in MWTW than in MSTP. Figure 3 g shows that the main contribution to acidification potential (AP) is also from the electricity consumption in both treatment plants with a 40% higher impact in MSTP. This is attributed to emissions of gases such as sulphur dioxide, sulphur monoxide and nitrogen oxides from fossil fuel combustion for electricity generation in Malaysia. Chemical consumption only accounts for 6.3% in MWTW and 0.1% in MSTP respectively. AP in dry season is 7.5% and 18.8% higher than a wet season in MSTP and MWTW, respectively.

From the LCA assessment above, it is found that a higher percentage of fossil fuel for electricity generation results in higher impacts in terms of categories of FEP, HTP, AP and ADFP. Therefore, moving the electricity generation from fossil fuels to renewable energy definitely benefits environment impact from WWTPs just as the UK did in the last few decades (UK Energy 2017). This is obviously a nation-level strategy on energy use and environmental protection. However, if WWTPs are able to recover energy from wastewater as much as possible to cover its own energy consumption, it will bring down environmental impacts in these 4 categories. For EP and GWP, they are more dependent on treatment performance and final effluent emission to the environment. More advanced treatment results in lower EP but higher GWP due to the increased chemical and energy consumption for advanced treatment. There is a trade-off between them. Meanwhile, the direct emission to GWP should not be neglected although it is still not common to be included in most studies on LCA. With regard to the seasonality effect by LCA analysis, it can be found that wet season in both plants has a less environmental impact than the dry season. This is mainly due to the dilution from storm runoff, thus the lower emission from effluent to the environment. In addition, less electricity is consumed to treat per 1 m^3^ wastewater during wet seasons due to the dilution of pollutants. MWTW shows a more obvious difference between two seasons while MSTP is more or less comparable except for EP category. From the raw sewage data, we can see that the strength of sewage to MWTW is much higher than that to MSTP, and the dilution during wet season plays a much obvious role in MWTW for reduced electricity consumption as well as reduced pollutant concentrations. Therefore, raw sewage strength is a key factor to lead to different environmental impact in the dry and wet seasons. This can well explain the contradictory results from the literature. Some studies reported lower environmental impact in a wet season than in dry season (Moreira et al. 2004; Mines et al. 2007; Joel et al. 2017), while Lorenzo-Toja et al. (2016) reported higher environmental impact in a wet season (winter). Therefore, it is very necessary to do LCA analysis with the consideration of rainfall effect on the sewage dilution especially when sewage has high strength to reflect real environmental impacts from WWTPs in different seasons. In addition, site-specific LCA for WWTP is also necessary to reflect the accuracy of environmental impact profile with different precipitation intensity (Yoshida et al. 2014).

#### Environmental impact of two WWTPs in wet and dry seasons using FU2

**Fig. 4:**
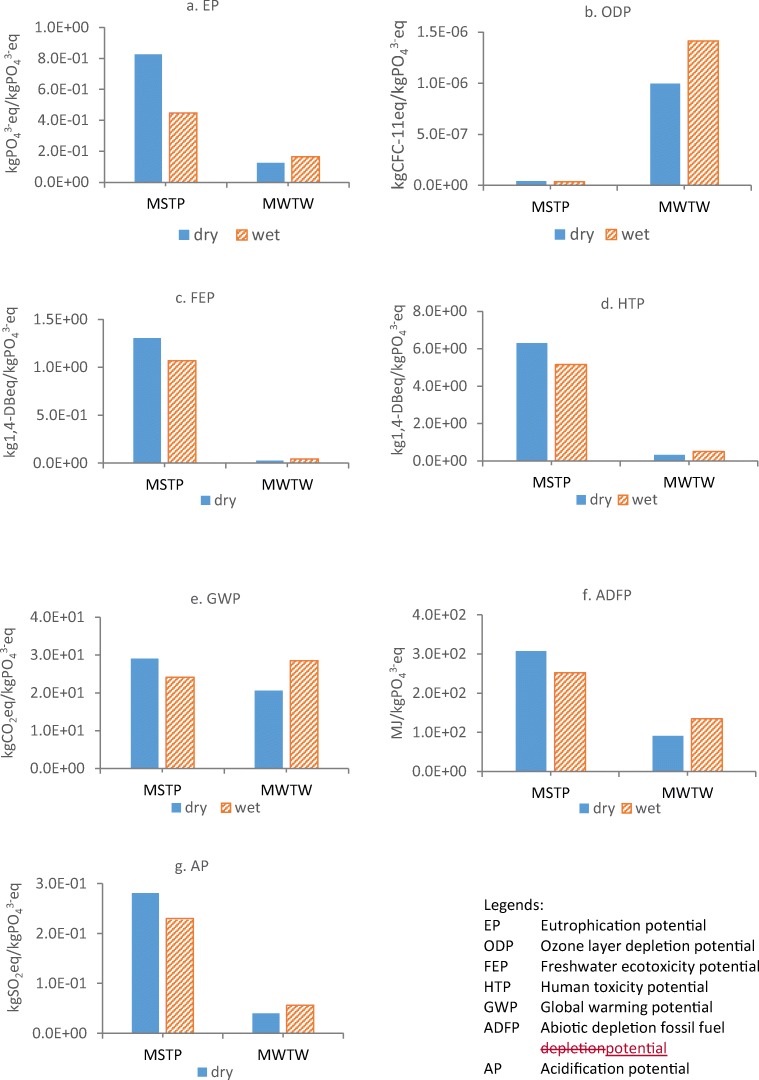
Environmental impact assessment in seven categories at Malaysian STP (MSTP) and Millbrook WTW (MWTW) in both dry and wet seasons by using FU of 1 kgPO_4_^3−^eq removed

Environmental impact analysis using FU2 was conducted to compare the impact difference of by using two functional units for both WWTPs in dry and wet seasons. It can be seen from Fig. 4 that except for ODP, MWTW still exhibits lower environmental impacts using FU2 compared to MSTP due to its high nutrient removal efficiency and better effluent quality. In MSTP, each environmental category in dry and wet seasons shows a similar trend when using FU1 and FU2. This suggests that no much difference is caused by adopting different functional units to WWTP with low strength wastewater. MWTW, however, demonstrates higher environmental impact in the wet season than the dry season with FU2, which is contrary to that by using FU1. For example, the lower EP from MWTW in the dry season using FU2 reflects a higher pollutant removal efficiency than in wet season, indicating that rainfall in wet season negatively affects wastewater treatment efficiency although it plays a dilution role. This result is in agreement with the study by Rodriguez-Garcia et al. (2014) who compared nitritation-anammox, nitrite shortcut and struvite crystallization processes for the supernatant treatment from anaerobic sludge digestion using two functional units, i.e. FU1 (per m^3^ treated wastewater) and FU2 (kg PO_4_^3^-eq removal). It was found that struvite crystallization process has the lowest eutrophication (EP) impact using FU1 due to the cleanest effluent (partially due to much lower influent pollutant concentrations) but the highest EP using FU2 due to the lowest removal of COD and N, and the least efficient in terms of EP reduction. In addition, a higher difference in all impacts ranging from 25 to 39% between dry and wet seasons is found by using FU2 compared to FU1.

It can be seen that, the selection of appropriate functional unit is prominent as the total treated water discharge volume to the environment is more in the wet season than the dry season, leading to the possible higher total pollutant mass load to the environment. It is noteworthy that although FU1 has been widely used for seasonal LCA assessment of WWTP (Piao and Kim 2016; Lorenzo-Toja et al. 2016; Li et al. 2017; Risch et al. 2018), effects from the variation of influent compositions and flow could not be reflected very well if only using per unit volume as a functional unit (Rodriguez-Garcia et al. 2011; Piao et al. 2016). The functional unit as per kgPO_4_^3^-eq removed based on eutrophying substances removal (e.g. COD, TN and TP) is believed to reflect the wastewater treatment performance better as pollutant removal from wastewater is the main objective of a WWTP to meet effluent limits by the legislation (Comas Matas 2012). Thus, considering pollutant removal efficiency during the wastewater treatment process, using FU2 as 1 kgPO_4_^3^-eq removed is more appropriate for an environmental impact assessment. It also makes the direct comparison between different WWTPs, or different seasons more meaningful as it is mainly based on pollutant removal by minimizing the effect from influent compositions and flows.

#### Detailed comparison of EP and GWP categories between FU1 and FU2

**Fig. 5:**
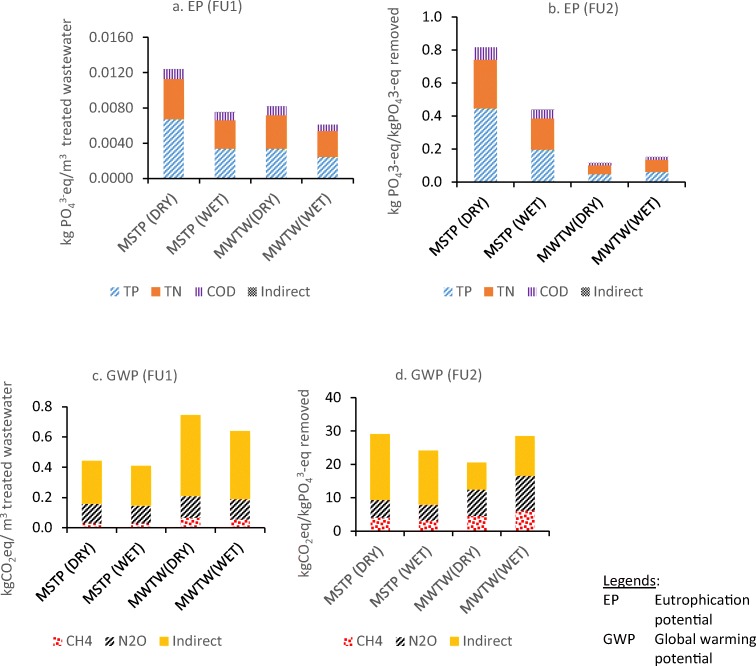
Comparison of eutrophication potential (EP) and global warming potential (GWP) at Malaysian STP (MSTP) and Millbrook WTW (MWTW) in the dry and wet seasons by using FU1 (per m^3^) and FU2 (per kgPO_4_^3^-eq removed), respectively

Based on Fig. 3, eutrophication (EP) and global warming potential (GWP) are two categories that are mostly affected by direct emissions from WWTPs. Therefore, they were further analysed to investigate the detailed contribution of each substance such as the contributions of COD, TN and TP to EP and, the contribution of CH_4_, and N_2_O to GWP. These could be further compared with indirect emissions from electricity and chemical consumption. As shown in Fig. 5, TP in the MSTP effluent contributed 54% in a dry season and 45% in a wet season to eutrophication category, respectively, with both functional units. TN is the second-highest contributor in MSTP with a contribution of 36% and 43% in a dry and wet season, respectively, followed by COD (11%) and negligible impact (< 1%) from the electricity and chemical consumption (indirect impact) in eutrophication. Rodriguez-Garcia et al. (2011) also highlighted the negligible impact of electricity and chemical consumption in this category. In MWTW, TN and TP present roughly comparable contributions to EP in both seasons using either FU. EP results by using FU2 show significantly lower EP in MWTW than MSTP due to considerable removal of pollutants including nutrients but MSTP does not have nutrient removal. This result suggests that FU2 reflects more effort made by the plant for pollutant/nutrients removal instead of the actual effluent emission only as FU1 does. The result from Piao and Kim (2016) also highlighted that their WWTP B using A2/O process with higher nutrient removal rate had a 30% lower EP impact compared to WWTP A with conventional activated sludge when using FU of 1 kg TN removed. The big difference of EP in dry and wet seasons in MSTP also suggests that nutrient removal in the dry season is more important than wet season to reduce EP.

For GWP category (Fig. 5c, d), MWTW has much higher GWP than MSTP using FU1 while GWPs in both plants are comparable by using FU2. This suggests again that FU selection is important for the comparison between different WWTPs with different influent compositions. The strength of wastewater to MWTW is higher than that to MSTP, resulting in almost double electricity consumption for treating per m^3^ wastewater. In addition, the additional chemical dose in MWTW for denitrification also contributes to GWP. Thus, it is plausible that GWP in MWTW is higher than MSTP when the comparison is based on per m^3^ treated wastewater. When the comparison is based on per kgPO_4_^3^-eq removed, however, it is found that MWTW is more environmentally efficient for the pollutant removal. This means that less environmental impact is caused by removing the same amount of pollutant. In addition, indirect contribution to GWP using FU2 is much smaller than using FU1 in MWTW, making the direct contribution from CH_4_ and N_2_O in treatment process more predominate (2 times more than that using FU1). Rodriguez-Garcia et al. (2014) also reported the higher percentage of direct emission to GWP with N-removal technology when using FU2, proving that direct emission could be dominant in the GWP impact category of WWTP. Nowadays, more on-site measurement of CH_4_ and N_2_O emission (Masuda et al. 2015; Schaubroeck et al. 2015; Piao et al. 2016) indicates that the direct emission of CH_4_ and N_2_O based on IPCC guidelines is underestimated. The direct GHG emission from a studied WWTP can contribute 75% to GWP with 53% from N_2_O and 22% from CH_4_ according to the average site-specific emission factor from the Korea Environmental Corporation Report 2008 (Piao et al. 2016). With 1 m^3^ treated water as FU, the emission of N_2_O and CH_4_ from Piao et al. (2016) is 3.5 and 5.5 times higher, respectively, than those in this study calculated based on IPCC guideline. The higher percentage of direct emission poses a great challenge to reduce GWP in WWTPs because currently there are still no widely accepted strategies, which can mitigate CH_4_ and N_2_O emissions effectively from wastewater treatment processes. In addition, the higher GWP in MWTW in wet season suggests a less efficient pollutant removal.

Overall, EP in MWTW is smaller than MSTP with both FUs due to the nutrient removal process. EP and GWP in MWTW in dry and wet seasons showed contrasting trends when using FU1 and FU2, respectively, indicating that MWTW is more sensitive to the selection of different functional units. This is probably stronger wastewater to MWTW with nutrient removal process, which is more affected by dilution from rainfall.

### Sensitivity analysis

Environmental impact assessment results in two plants highlight that nutrients in effluent and electricity consumption are the major factors to affect environmental impacts. Table 5 shows how environmental impacts in MSTP and MWTW in the dry season were affected by varying ± 10% of selected inventory component values s such as nutrient concentrations and electricity consumption or chemical consumption. Data from FU1 was selected for this analysis to facilitate the comparison with the results with other studies. Environmental impact categories such as FEP, HTP, GWP, ADFP and AP varied from ± 7.4 to ± 9.9% to respond to the change in electricity consumption by ± 10%. The response to ± 10% change in electricity consumption in MWTW was even less obvious, ranging from ± 2.8 to ± 9.5% in six categories except for eutrophication. The less sensitivity to electricity consumption values in MWTW is mainly due to lower fossil fuel percentage used for electricity generation in the UK compared with Malaysia (Table S2 in the supplementary). This result is in agreement with Piao et al. (2016) that electricity consumption caused the most sensitive change to acidification and human toxicity in all WWTPs studied. Eutrophication (EP) changed by ± 9.1% in MSTP and by ± 8.5% in MWTW to respond to ± 10% change in TP and TN concentrations in the effluent while the other six categories are almost unaffected. Finally, the chemical consumption shows less effects on all the categories with the highest FEP change by ± 6.1%. In general, the variation of electricity and nutrients in the effluent by 10% will not cause an environmental impact change more than 10%, suggesting a less sensitivity of environmental impact results to inventory data.

**Table 5:** Sensitivity analysis results by changing selected inventory data by ± 10% of in Malaysian STP and Millbrook WTW in the dry season according to 1 m^3^ of treated wastewater

	MSTP (%)		MWTW (%)		
Inventory components	Electricity consumption	TN and TP in the effluent	Electricity consumption	TN and TP in the effluent	Chemical Consumption*
Eutrophication EP	± 0.50	± 9.05	± 0.10	± 8.50	± 0.02
Ozone layer depletion (ODP)	± 0.06	± 0.01	± 9.52	± 0.00	± 0.10
Fresh water ecotoxicity FEP	± 9.95	± 0.00	± 2.78	± 0.01	± 6.11
Human toxicity HTP	± 9.88	± 0.00	± 5.56	± 0.00	± 2.86
Global warming potential GWP	± 7.39	± 0.00	± 5.27	± 0.02	± 2.05
Abiotic depletion (fossil fuels) ADFP	± 9.79	± 0.00	± 7.73	± 0.00	± 1.87
Acidification AP	± 9.95	± 0.00	± 9.10	± 0.00	± 0.62

TN and TP = total nitrogen and total phosphorus

*Sensitivity analysis on chemical consumption was not conducted in MSTP since chemical consumption contributes less than 1% to all environmental impacts categories

## Conclusions

The influence of rainfall on the environmental impacts of two large centralized WWTPs with different wastewater strengths and sewer systems but similar rainfall effects on influent flow rate was investigated by using LCA in this study. Meanwhile, two different functional units were evaluated to see how the selection of functional units affect LCA results in the circumstance of rainfall effects. The results are summarized as below:

- The coefficients between monthly rainfall and the influent flow rate are similar at around 2500 m^3^ influent flow rate/mm precipitation although two WWTPs have different sewer systems and wastewater strengths. This disclose that rainfall intensity affects the quantity and quality of influent to WWTPs, but the extent of effect is not directly determined by rainfall intensity or sewer system, i.e. if it is a combined or a separate sewer system.
- Based on the life cycle analysis from two large centralized WWTPs, nutrients in effluent and electricity consumption are the major factors to affect the environmental impacts, while chemical consumption and transportation has minimal impact on the environment due to the little consumption of chemicals.
- When per m^3^ treated wastewater was used as the functional unit, all environmental impact categories in MSTP except eutrophication potential are almost similar in dry and wet seasons while MWTW shows higher environmental burdens in the dry season than a wet season for all seven environmental impact categories.
- When per kgPO_4_^3^-eq. removed was used as the functional unit, all seven environmental impacts in MWTW showed higher values in the wet season than the dry season, while the selection of either of functional units has no influence on the environmental impact categories in MSTP.

The results from this study demonstrate that rainfall effects on the environmental impact of WWTPs are more effective in MWTW with higher wastewater strength. The contrasting results of environmental impacts in MWTW during wet and dry seasons by using two different functional units suggest that the selection of functional unit is dependent on the comparison purpose, such as the impact of WWTPs effluent to the environment only, or the combined effects from effluent and WWTP treatment efficiency. This work identified the importance of wastewater strength and functional units to the studies of rainfall effects on the environmental profile of WWTPs, which could serve as a basis for further rainfall studies with different coefficients between rainfall intensity and inflow rate, advanced treatment and others. In addition, the environmental impact assessment in this study provides guidance for a better eutrophication potential control especially in vulnerable receiving waters in different seasons.

## Electronic supplementary material

ESM 1 (DOCX 36 kb)
