# Supplementary material for: Assessing environmental impacts of large centralized wastewater treatment plants with combined or separate sewer systems in dry/wet seasons by using LCA
Source: Environ Sci Pollut Res Int. 2020 Feb 20;27(13):15674–90. doi: 10.1007/s11356-020-08038-2 (PMC7190677; doi:10.1007/s11356-020-08038-2)
Supplement: Supplementary file 1 — (DOCX 36 kb) [file 11356_2020_8038_MOESM1_ESM.docx]

# Supplementary Information

**Assessing environmental impacts of large centralized wastewater treatment plants with combined or separate sewer systems in dry/wet seasons by using LCA**

**Siti Safirah Rashid ^a^, Yong-Qiang Liu ^a^***

*^a^ Faculty of Engineering and Physical Sciences, University of Southampton, Southampton, SO17 1BJ, United Kingdom*

*Corresponding author. Tel.: +44 02380592843

*E-mail address*: [Y.Liu@soton.ac.uk](mailto:Y.Liu@soton.ac.uk)

**Table S1**

Pearson correlations between average monthly sewage temperature, rainfall, the inflow of wastewater, power consumption and effluent pollutant concentrations at Malaysian STP and Millbrook WTW

| MSTP | temperature | rainfall | inflow | power | BOD_5_ | COD | TN | TSS |
| --- | --- | --- | --- | --- | --- | --- | --- | --- |
| temperature | 1.00 |  |  |  |  |  |  |  |
| rainfall | 0.02 | 1.00 |  |  |  |  |  |  |
| inflow | 0.21 | 0.84 | 1.00 |  |  |  |  |  |
| power | 0.33 | 0.54 | 0.44 | 1.00 |  |  |  |  |
| BOD_5_ | -0.23 | -0.23 | -0.39 | -0.10 | 1.00 |  |  |  |
| COD | -0.35 | -0.02 | -0.32 | 0.08 | 0.77 | 1.00 |  |  |
| TN | -0.75 | -0.28 | -0.36 | -0.79 | 0.19 | 0.12 | 1.00 |  |
| TSS | -0.32 | -0.35 | -0.53 | -0.30 | 0.53 | 0.75 | 0.37 | 1.00 |
| MWTW |  |  |  |  |  |  |  |  |
| temperature | 1.00 |  |  |  |  |  |  |  |
| rainfall | -0.19 | 1.00 |  |  |  |  |  |  |
| inflow | -0.39 | 0.63 | 1.00 |  |  |  |  |  |
| power | 0.57 | 0.16 | 0.14 | 1.00 |  |  |  |  |
| BOD_5_ | 0.62 | -0.61 | -0.50 | 0.49 | 1.00 |  |  |  |
| COD | 0.34 | 0.30 | -0.02 | 0.38 | 0.08 | 1.00 |  |  |
| TN | 0.25 | 0.19 | -0.16 | 0.04 | 0.07 | 0.38 | 1.00 |  |
| TSS | 0.04 | 0.29 | 0.01 | 0.30 | 0.04 | 0.54 | 0.05 | 1.00 |

**Table S2**

The national electricity generation mix in Malaysia and United Kingdom

| Energy source | Malaysia (%)^a^ | United Kingdom (%)^b^ |
| --- | --- | --- |
| Natural gas | 45 | 41 |
| Coal | 41 | 11 |
| Oil | 7 | - |
| Renewable energy (incl. biomass, wind, solar) | 7 | 27 |
| Nuclear | - | 15 |
| Others (i.e interconnector) | - | 6 |

^a^ Source: Ecoinvent v3.3

^b^ Source: Ecoinvent v3.3 and www.mygridgb.co.uk

**Table S3**

Life cycle inventory (LCI) data of Malaysian STP and Millbrook WTW per functional unit 2 (eutrophication reduction – 1 kgPO_4_^3-^eq).

| Inventory components | Malaysian MSTP | | Millbrook MWTW | | Unit/ kgPO_4_^3-^eq |
| --- | --- | --- | --- | --- | --- |
|  | Dry season^A^ | Wet season^B^ | Dry season (summer)^C^ | Wet season (winter)^D^ |  |
| 1.Electricity consumption | 1.80E+01 | 1.47E+01 | 9.15E+00 | 1.28E+01 | kWh |
| 2.Transportation of sludge and waste | 4.52E-01 | 3.99E-01 | 4.03E-01 | 6.91E-01 | t.km |
| Polymer consumption |  |  |  |  |  |
| 3.Methanol | - | - | 5.40E-02 | 9.27E-02 | kg |
| 4.Polyelectrolyte | 3.60E-02 | 3.18E-02 | 1.06E-01 | 1.83E-01 | kg |
| 5.Lime | - | - | 1.22E+00 | 2.10E+00 | kg |
| Emission to air |  |  |  |  |  |
| 6.Methane, CH_4_ | 1.46E-01 | 1.15E-01 | 1.64E-01 | 2.20E-01 | kg |
| 7.Dinitrogen monoxide, N_2_O | 2.04E-02 | 1.80E-02 | 3.00E-02 | 3.96E-02 | kg |
| Emission to water |  |  |  |  |  |
| 8.Total COD | 3.37E+00 | 2.51E+00 | 6.55E-01 | 8.00E-01 | kg |
| 9.Total nitrogen | 7.00E-01 | 4.47E-01 | 1.26E-01 | 1.73E-01 | kg |
| 10.Total phosphorus | 1.46E-01 | 6.44E-02 | 1.57E-02 | 2.00E-02 | kg |

^A^ From January to March 2016; ^B^ From September to November 2016

^C^ From June to July 2017^; D^ From January to February 2017

**Fig. S1**. The comparison of average monthly rainfall data for Malaysian STP in the year 2016 (from Malaysian Meteorological Department) and average rainfall data from the year 2010 to 2016 with standard deviation (from international weather website : [www.worldweatheronline.com](http://www.worldweatheronline.com)). Dry season was identified from January to March while the wet season was from September to November in Penang, Malaysia

**Fig. S2**. The comparison of average monthly rainfall data for Millbrook WTW in the year 2017 and average rainfall data from the year 2013 to 2017 with standard deviation (from international weather website: [www.worldweatheronline.com](http://www.worldweatheronline.com)). Winter (wet) was identified in January and February while summer (dry) was in June and July in Southampton, UK

**Fig. S3**. The comparison among dry season, wet season and combine sewer overflow (CSO) occasion in terms of Rseven impact categories in Millbrook WTW using function unit (FU1)
